# Construction and Simulation of High-Quality Development of China’s Resource-Based Cities Driven by Innovation Based on System Dynamics

**DOI:** 10.3390/ijerph20064812

**Published:** 2023-03-09

**Authors:** Shuai Liu, Guoxin Jiang, Le Chang, Chao Huang

**Affiliations:** 1 School of Marketing Management, Liaoning Technical University, Huludao 125105, China; 2 School of Business Administration, Liaoning Technical University, Huludao 125105, China

**Keywords:** resource-based cities, high-quality development, innovation-driven, system dynamics, ecological environment

## Abstract

Innovation is the primary driving force for development; the high-quality development of resource-based cities is ultimately driven by innovation. We constructed an innovation-driven high-quality development system for resource-based cities, including resource, economic, social, and environmental subsystems; according to the interaction between the internal elements of each subsystem, a dynamic model of the innovation-driven high-quality development system of resource-based cities was established, and we selected policy adjustment variables to simulate six policy scenarios. Thus, we simulated high-quality development trends from 2008 to 2035. The results indicate that the policy scenario of increasing innovation investment can promote high-quality development; the policy of increasing innovation investment has a significant effect on economic growth, while it damages the urban ecological environment, and the ideal policy scenario is the environmental priority mode, which appropriately increases innovation investment and reasonably allocates it within the system.

## 1. Introduction

Resource-based cities are cities where the main industries are the exploitation and processing of local natural resources, such as minerals or forests. With the endowment of resources, they provide abundant materials, funds, and talent for the country and make a significant contribution to the promotion of the healthy development of the national economy. Since the 1980s, China has accelerated the process of industrialization, and the overexploitation of resources has led to mine exhaustion and urban decline in many resource-based cities. Due to the exhaustion of the resources on which industrial development depends, the urban industrial chain has broken down, and it has experienced major problems, such as a lag in the development of successive alternative industries, an insufficient employment of labor, and the destruction of the ecological environment. The situation is becoming increasingly serious due to the imbalance and lack of coordination between resource development and economic development, social stability, and environmental protection; it is very important to solve this problem for governments.

China’s development has entered a new era, transitioning from a high-speed growth stage to a high-quality development stage and from a factor-driven stage to an innovation-driven stage. Innovation is the first driving force leading to development. The realization of high-quality economic development depends on innovation. In innovation-driven terms, “drive” refers to the initiative to promote economic growth. It refers to the development mode with innovation as the main driving force, realizing a new combination of advanced innovation elements, such as knowledge, information, management, and technology, and the reallocation process of production elements from the old low-income combination to the new high-income combination. The economic structure is continuously optimized, and the mode of economic development is gradually transformed; thus, high-quality development will be achieved [1]. This is an essential strategy for resource-based cities to achieve high-quality development, that is, building a high-quality growth system driven by innovation and adjusting the allocation structure of the system elements.

At present, the research on the high-quality development of resource-based cities mainly focuses on transformation development, sustainable development, and coordinated development. Studying the relationship between the innovation-driven and high-quality development of resource-based cities will help to identify new economic growth points and provide a theoretical basis for the high-quality development of resource-based cities. Does innovation drive the high-quality development of resource-based cities? What is the mechanism of innovation in driving the high-quality development of resource-based cities? How should resource-based cities implement innovation-driven policies to achieve high-quality development?

Against this background, the present study constructs a high-quality development system dynamic model of China’s resource-based cities and simulates the effect of high-quality development policies from the perspective of innovation. The findings can provide insights into the direction of policy decisions and are conducive to accelerating the high-quality development process of China’s resource-based cities.

This article is composed of six sections. Their organization is as follows: Section 1 is the introduction, and it mainly considers the research background and significance. Section 2 is a literature review. Section 3 presents the methodology and methods, and it considers the study area, research methods, and data. Section 4 presents the main results. Section 5 is the discussion; it discusses the results and the deficiencies of this study. Section 6 is the conclusions and policy implications; it presents the conclusions of the research and policy implications.

## 2. Literature Review

In 1921, the concept of a mining town was put forward by Auronsseau [2]. Subsequently, scholars have studied the economic development of resource-based cities from different perspectives and achieved rich results. Lucas [3] divided the development of resource-based towns into the construction period, the development period, the transition period, and the maturity period, and Bradbury [4] added the recession stage and the closure stage. Markey [5] proposed that resource-based cities should change from economic and resource advantage to competitive advantage cities. Lockie et al. [6] used the life cycle theory to predict that the development cycle stage of resource-based cities belongs to the “M” type. Barnes et al. [7] pointed out that industrial transformation benefits the development of resource-based cities. At the same time, Arouri et al. [8] proposed that the transformation of resource-based cities needs to rely on their own natural resource endowment to improve their urban functions. Peng et al. [9] evaluated different planning schemes for industrial change, and Ansari et al. [10] proposed that labor outflow from resource-exhausted areas could easily lead to a resource curse in other regions. In general, western scholars mainly study the transformation of resource-based cities from the perspectives of the mining area development life cycle, industrial upgrading, and industrial structure adjustment, and this has important theoretical and practical significance.

In the 21st century, research on the high-quality development of resource-based cities has focused mainly on transformation and sustainable development [11,12]. Green transformation provides solutions and action guidelines for the high-quality development of resource-based cities [13]. The driving force for the transformation of resource-based cities comes from innovation, which is reflected in scientific and technological innovation, technological innovation, and government regulation [14,15,16,17]. Other scholars, such as Zeng, Wan et al., put forward suggestions for the transformation of resource-based cities from the perspective of innovation-driven development [18,19,20]. Lu et al. [21] showed that an improvement in the level of openness to the outside world was important for the new period to promote the high-quality transformational development of resource-based cities. It can be seen that scholars believe that the transformation of resource-based cities can be achieved through innovative, green, open, and other perspectives.

Guo et al. [22] empirically analyzed the impact of resource efficiency and environmental efficiency on the sustainable development of resource-based cities. The guaranteed strategy for the sustainable development of resource-based cities must change from the policy path to the legal one [23]. The sustainable development of resource-based cities should be supported by the science and technology industry, and technological innovation is an inevitable choice for the sustainable development of coal-resource-based cities [24]. It can promote the extension of the industrial chain with the help of technological innovation and high technology for the sustainable development of resource-based cities [25,26,27,28]. The sustainable development of resource-based cities cannot be separated from the support of the environment, policy, law, science and technology, and other fields. The role of innovation in the transformation of resource-based cities has been affirmed by most scholars, but there are few studies that verify the impact of innovation factors on the transformation and upgrading of resource-based cities.

As the review of the literature demonstrates, many studies have made great achievements in the innovation, transformation, and sustainable development of resource-based cities, which has laid an important foundation for this study. This paper contributes to the following aspects: Firstly, the literature is mainly focused on calculating the quantitative indicators or describing the structure and analyzing the factors influencing the high-quality development of resource-based cities based on the macroenvironment outside the system; meanwhile, a comprehensive analysis of the innovation-driven high-quality development of resource-based cities is lacking. This paper comprehensively and systematically studies and explains the mechanism of the innovation-driven high-quality action of resource-based cities, combining microfactors with macrofactors. Secondly, this paper analyzes the internal mechanism of high-quality development driven by innovation in resource-based cities. Based on system thinking, an innovation-driven high-quality development system for resource-based cities is constructed, and the interaction among various elements is analyzed by creating a system dynamics model. Thirdly, this paper simulates the future development trend of the high-quality development system of resource-based cities under different policy scenarios by adjusting the policy parameters to identify and evaluate governance results, which can provide theoretical support for resource-based cities to formulate high-quality development policies driven by innovation.

## 3. Methodology and Data

A system dynamics model can reveal the non-linear relationships among the factors and the multiple feedback mechanisms within the system [29,30]. It simulates the actual scenario on a computer using a simulation language and utilizes qualitative and quantitative analyses to reveal the complex, dynamic relationships among subsystems, providing an effective method to solve the complex problem of dynamic relationships among the structure, function, and behavior of the system. Thus, we adopted the system dynamics method to build a simulation of a system for the innovation-driven high-quality development of resource-based cities; this simulation aims to describe the internal structure, function, and behavior of the system, providing an effective policy simulation analysis tool for governments.

### 3.1. System Boundary

We study the complex and non-linear system of the innovation-driven high-quality development of resource-based cities, which contains many factors. The National Sustainable Development Plan of Resource-Based Cities (2013–2020) proposed the sustainable development of resource-based cities from the perspectives of the economy, people’s livelihoods, resources, and environmental protection [31]. Zeng et al. [24,32,33] described a transformation system of resource-based cities from the perspectives of resources, economy, environment, population, and society. The innovation-driven high-quality development of resource-based cities refers to the rational allocation of resource elements driven by innovation. By providing advanced technology, equipment, and raw materials, the efficiency of the use of innovation elements is effectively improved, which is conducive to transforming innovation achievements into progressive productive forces, promoting the level of economic development of resource-based cities, improving the utilization rate and recovery rate of resources, reducing waste emissions and pollution to the ecological environment in the production and living processes of resource-based cities, alleviating population loss and unemployment, and finally realizing high-quality development. In the system, innovation elements are used as catalysts, and resources, technologies, talent, and the environment are exchanged to produce interrelated and interactive relationships, so it is impossible to separate a specific factor from the system and study it alone. Therefore, considering the representativeness and availability of existing research results and data, it is finally determined that the innovation-driven high-quality development system of resource-based cities is a complex and non-linear dynamic system that comprises four subsystems, resource, economic, social, and environmental subsystems, as shown in Table 1.

Resources are the material basis of urban development; the resource subsystem examines the impact of resource consumption on high-quality urban development. The economy is the foundation of the social development of resource-based cities and is also the primary source of resource consumption and environmental pollution. The economic subsystem shows the level of economic development of resource-based cities driven by innovation. The purpose of the high-quality development of resource-based cities is to improve people’s livelihoods and promote all-round social progress. The social subsystem indicates the population, education, and social progress in the high-quality development of resource-based cities. The environment provides material energy for resource-based towns, as well as the final site for the discharge of urban waste. The environmental subsystem shows the impact of the high-quality development of resource-based cities on the environment.

### 3.2. System Causal Loop Diagrams

**Figure 1:**
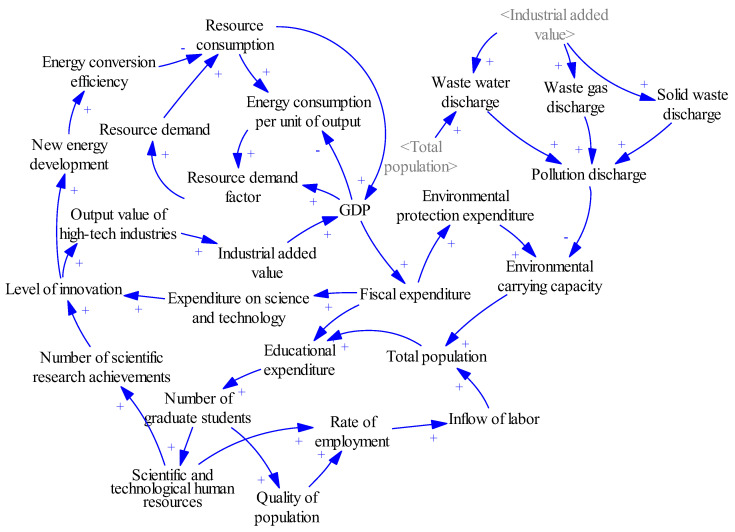
System causal loop diagrams of innovation-driven high-quality development of resource-based cities.

Depending on the structure of the system and the relationships between subsystems, the causal relationship in an innovation-driven high-quality development system of resource-based cities is described using Vensim-PLE 8.0.4 software, as shown in Figure 1.

### 3.3. Model Construction and Test

#### 3.3.1. City Selection

**Figure 2:**
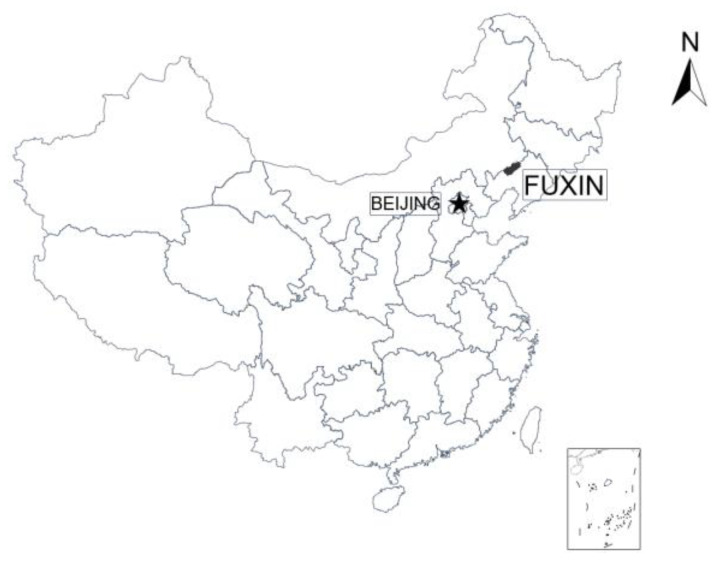
Location of Fuxin in China.

Fuxin City, Liaoning Province, is rich in coal resources, and it is one of the earliest energy bases in China (Figure 2). With the depletion of coal mining in Fuxin City in 2001, it was recognized as a pilot city for the transformation of resource-based cities in China. Thus, Fuxin City has begun exploring urban transformation and has achieved good results. From 2003 to 2012, Fuxin’s economic development continued to improve, with an average annual GDP growth rate of 21%. After China entered the new normal, Fuxin’s economic growth slowed down. Obviously, from 2013 to 2018, the average annual GDP growth rate dropped to approximately 3%, the industrial added value decreased year by year, the labor outflow was severe, and the contradictions in economic and social development became increasingly prominent. Since 2018, Fuxin City has put forward the requirement of high-quality development to promote the quality and growth of economic development. Therefore, taking Fuxin as an example, it is considered a representative in the study of the high-quality development of China’s resource-based cities.

#### 3.3.2. System Flow Diagram

**Figure 3:**
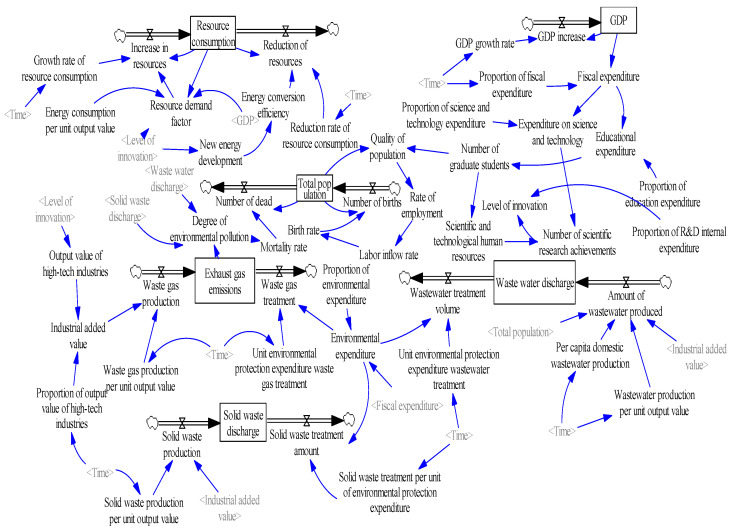
System flow diagram of innovation-driven high-quality development of resource-based cities.

Considering the causal relationship of the system and according to the role of each element of the high-quality development system of innovation-driven resource-based cities, a flow diagram model is constructed using Vensim-PLE 8.0.4 software, as shown in Figure 3. There are 53 variables in the model, including 6 state variables, 11 rate variables, 19 auxiliary variables, and 17 constants. The data come from the Fuxin Statistical Yearbook, the Fuxin National Economic and Social Development Statistical Bulletin from 2008 to 2018, etc., which are reliable, accurate, and standardized. The relationship equation between variables is mainly constructed using fundamental functional relationships, and the parameters involved are obtained using an average arithmetic method according to the existing data. For some data with missing statistical values, a regression analysis is used to deduce the equation. The table function is used to construct the equation if the relationship between variables is not apparent.

#### 3.3.3. Model Test

In order to ensure that the SD model can accurately simulate the actual condition, the suitability and authenticity of the model were tested. Based on the data from Fuxin in 2008, this study simulated the economic development and operation of Fuxin from 2008 to 2018 to verify the structure, equations, and units of the model and test the sensitivity and historicity simultaneously. The historical results of the resource consumption and total population tests are listed in Table 2. The table shows that the relative errors of the simulation results are less than 9%, which is within the acceptable range, indicating that the model has a rather high degree of fit to the real high-quality development of the resource-based city. Thus, it can be used for simulation.

### 3.4. Scenario Design

The innovation-driven high-quality development system of resource-based cities is a complex system that integrates the resource, economic, social, and environmental subsystems and needs the cooperation of stakeholders, such as governments, enterprises, universities, and research institutes. The resources within the system depend not only on the market mechanism to achieve optimal allocation but also on the government to formulate a scientific and efficient policy combination to guide the rational flow of resources. According to the economic operation of Fuxin City, considering the variables in the SD model, the energy consumption per unit output value, the proportion of R&D internal expenditure, the proportion of science and technology expenditure, the proportion of education expenditure, and environmental protection expenditure are selected as policy adjustment variables, which are considered within the resource, economic, society, and environmental subsystems, respectively. The energy consumption per unit output value is calculated for industrial enterprises above the designated size, and the proportion of R&D internal expenditure refers to the proportion of R&D internal spending to the GDP of industrial enterprises above the designated size; the other three variables refer to the proportion of fiscal spending.

To analyze the policy effect of the innovation-driven high-quality development system of resource-based cities and the influence of different policy scenarios, six scenarios are designed, as shown in Table 3. Regarding the energy consumption per unit output value, based on the China Statistical Yearbook 2019, it is approximately 0.000052 tons of standard coal per CNY 10,000. Other parameter values are set according to the current economic development situation of Fuxin City to analyze the influence of different intensities of policy implementation on the high-quality development of resource-based cities.

The baseline scenario is the current implementation policy of Fuxin’s economic development. The parameter value is the mean value of Fuxin from 2008 to 2018. The energy consumption per unit output value is 0.000078 tons of standard coal per CNY 10,000, which is higher than the national average. With the exception of some years, the parameter value for most years is stable in the long-term development process, so the mean value is taken as the baseline scenario.

Based on the baseline scenario, the steady development mode reduces the energy consumption per unit output value to the national average level and raises the other parameter values by a certain proportion. It is necessary to reduce the dependence on resources and energy to reflect the innovation-driven high-quality development of resource-based cities. All types of enterprises should increase their R&D expenditure to produce more innovative achievements. Government departments should increase their investment in science, technology, and education to promote the quantity and quality of scientific and technological achievements and high-level talent in resource-based cities, and they should also increase their investment in environmental protection to improve environmental pollution so as to realize the high-quality development of innovation-driven resource-based cities through the cooperation of various stakeholders.

Compared with the steady development mode, the technology-driven mode increases the proportion of R&D internal expenditure and science and technology expenditure. The “14th Five-Year Plan” suggests that innovation-driven development should strengthen the leading position of companies in innovation, and companies and the government should increase their investment in research and development [34]. Therefore, to achieve high-quality growth in resource-based cities, the government and enterprises will increase their investments in science and technology.

Compared with the steady development mode, the education priority mode increases the proportion of education expenditure. The “14th Five-Year Plan” suggests that innovation-driven development should stimulate the innovation vitality of talent and cultivate and introduce talent [34]. Resource-based cities must take talent as the first resource, and the government must increase its investment in talent training to increase the proportion of education expenditure as a possibility for policy change.

The environmental priority mode reduces energy consumption per unit output value and increases the proportion of environmental protection and education expenditure compared to the steady development mode. The Guiding Opinions on Strengthening Classification Guidance and Cultivating New Kinetic Energy for Transformation and Development of Resource-Based Cities propose to accelerate the improvement of the environmental protection service system and strengthen pollution control, energy conservation, and emission reduction [35]. Most resource-based cities have the problem of the insufficient development of new industries, leading to a decrease in talent attraction and a severe lack of talent in cities. The government needs to formulate active talent policies to attract and retain talent, and increasing the proportion of education expenditure is the essential embodiment of talent policies. Therefore, resource-based cities can intensify the adjustment of policies via these three indicators.

The accelerated development mode increases the adjustment of all the parameter values, which are higher than those in the steady development mode. The policy implication is that, in order to achieve the high-quality development of resource-based cities, the government, enterprises, and other stakeholders should vigorously increase their investments in innovation, education, and environmental protection and analyze the impact of policy intensity improvement on the high-quality development of resource-based cities driven by innovation.

## 4. Results

**Figure 4:**
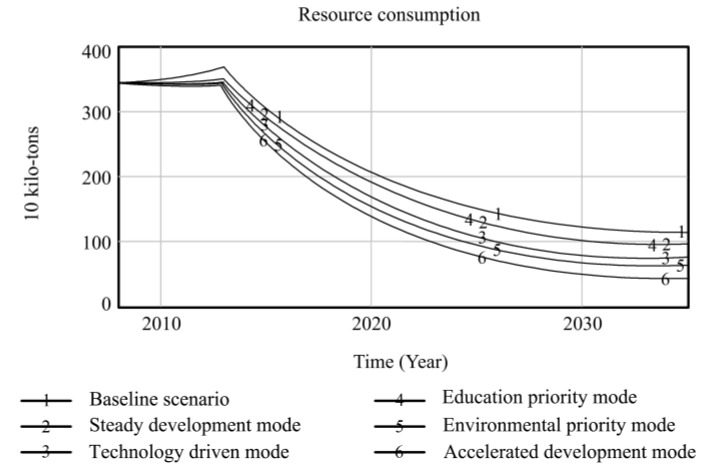
Simulation curve of energy consumption in Fuxin from 2008 to 2035.

**Figure 5:**
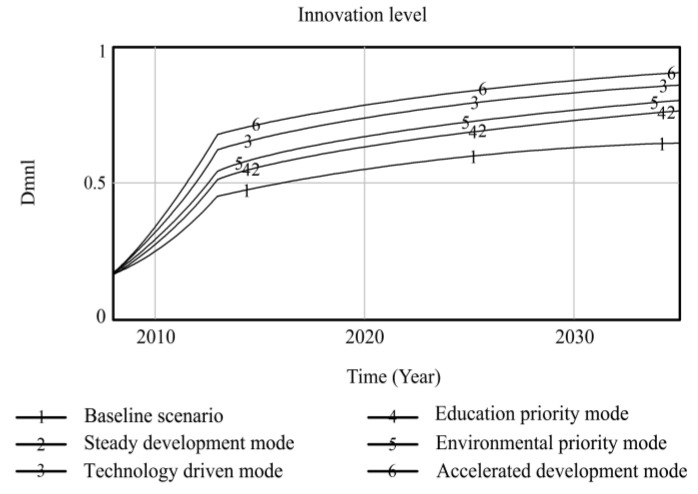
Simulation curve of innovation level in Fuxin from 2008 to 2035.

**Figure 6:**
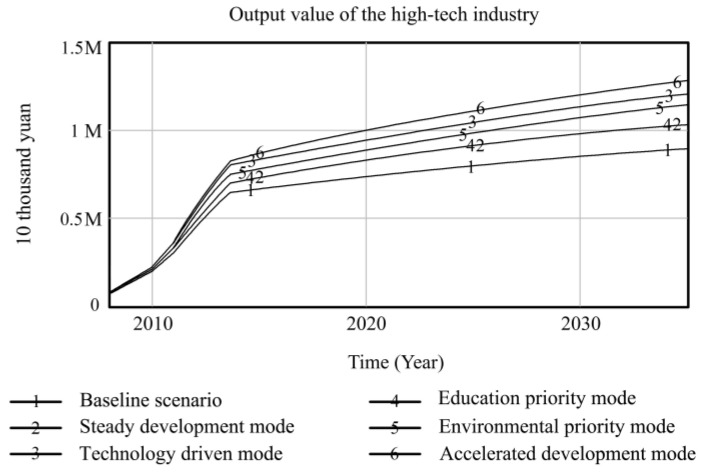
Simulation curve of the high-tech industrial output value in Fuxin from 2008 to 2035.

**Figure 7:**
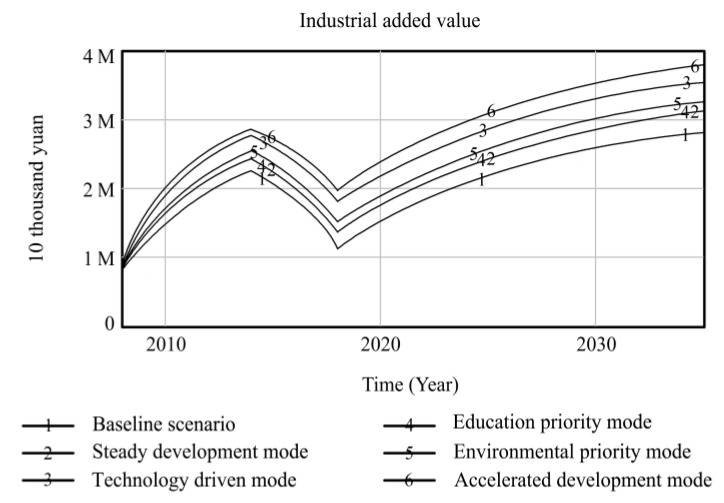
Simulation curve of industrial value in Fuxin from 2008 to 2035.

**Figure 8:**
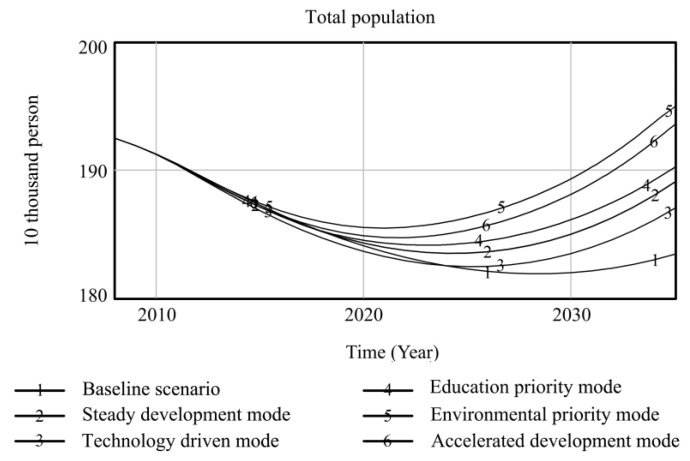
Simulation curve of population in Fuxin from 2008 to 2035.

**Figure 9:**
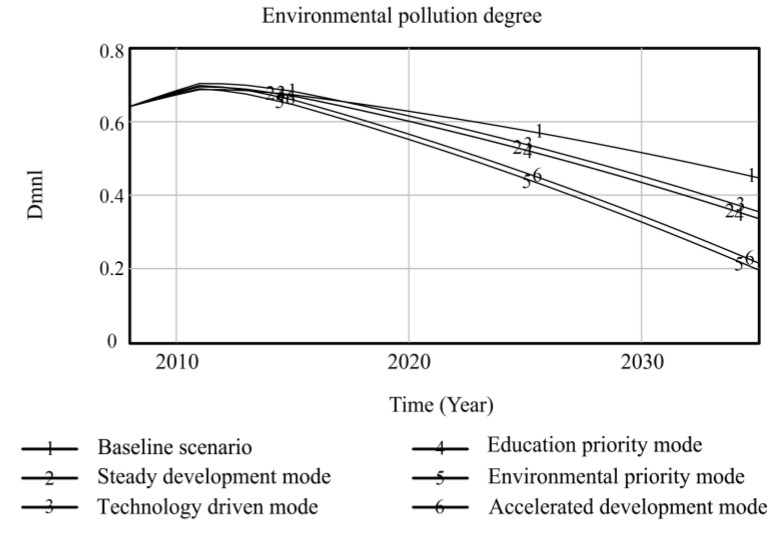
Simulation curve of environmental pollution in Fuxin from 2008 to 2035.

Using Vensim-PLE software, the high-quality development system of resource-based cities driven by innovation is simulated and predicted. Taking the 2008 data as the original values, the resource consumption, innovation level, output value of high-tech industries, industrial added value, total population, and degree of environmental pollution are selected as the main observation variables, and other variables are used as auxiliary explanatory variables. Resource consumption reflects the dependence on resources in the high-quality development process of resource-based cities. The level of innovation includes internal R&D expenditure and the proportion of science and technology expenditure, which comprehensively reflects the long-term development of innovation in resource-based cities. The output value of high-tech industries and the added value of the industry are used as indicators to measure the economic output of the innovation-driven high-quality development of resource-based cities. The total population is used as a social benefit indicator to measure the innovation-driven high-quality development of resource-based cities. The degree of environmental pollution is composed of solid waste emissions, waste gas emissions, and wastewater emissions, reflecting the environmental situation of the innovation-driven high-quality development of resource-based cities. Based on the six policy scenarios, the high-quality development trend of innovation-driven resource-based cities from 2008 to 2035 is simulated. The simulation results of the observation variables are shown in Figure 4, Figure 5, Figure 6, Figure 7, Figure 8 and Figure 9, and the average annual growth rates of the observation variables from 2008 to 2035 are shown in Table 4.

Firstly, in the baseline scenario, all the simulated values of the six observation variables are the maximum or minimum values. The level of urban innovation, the total population, the output value of high-tech industries, and the added value of the industry are lower than the corresponding values in the other five policy scenarios. The degree of resource consumption and environmental pollution is higher than that in the other five policy scenarios. This indicates that the level of high-quality development of resource-based cities in the baseline scenario is low, so it is necessary to explore the optimal input–output ratio of the innovation-driven high-quality development system optimization policy model to achieve the high-quality development of resource-based cities.

Secondly, the observation variables’ values in the steady development mode are at the moderate level, which is improved compared with those in the baseline scenario. Reducing energy consumption per unit output value and increasing investment in environmental protection steadily reduce resource consumption and environmental pollution in resource-based cities. When the environment in resource-based cities is improved, the proportion of education expenditure increases, and the total population of resource-based cities increases significantly under the joint action of the two observation variables. The increase in internal R&D spending and science and technology expenditure promotes the level of innovation of resource-based cities, which drives the output and industrial added value of high-tech industries to rise. This shows that governments can effectively promote the high-quality development of resource-based cities by increasing its investment in policies and guiding the government, enterprises, and universities to actively participate in innovation.

Thirdly, the technology-driven mode increases the proportion of R&D internal expenditure and science and technology expenditure based on the steady development mode, which promotes the innovation level of resource-based cities to be significantly improved and drives the rapid growth of economic output indicators, such as high-tech industrial output value and industrial added value. The values of the three observation variables are at a high level in all scenarios. Under the combined action of improving the level of innovation and reducing the energy consumption per unit of output value, the consumption of urban resources is lower than that in the former two scenarios. Although the proportion of environmental protection expenditure is increased due to the rapid development of the urban industry, the ecological bearing pressure is too high to some extent, resulting in high environmental pollution. The negative feedback effect of environmental pollution accelerates an increase in the mortality rate, while the positive feedback effect of education expenditure promotes an increase in the birth rate. The combined effect of the two makes the total population of resource-based cities slightly increase compared to the baseline scenario. It can be seen that increasing investments in science and technology to promote high-quality development in resource-based cities can drive various economic outputs and reduce resource consumption. It is necessary to coordinate the relationship between economic growth and the ecological environment to avoid adverse effects on the urban environment.

Fourthly, the high education expenditure in the education priority mode has no significant impact on the observation variables of the high-quality development of resource-based cities. Compared to the steady development mode, the observation variables, except for the total population, do not change significantly. This shows that talent is an essential resource for the innovation-driven high-quality development of resource-based cities. However, the policies designed by governments to train talent, such as increasing education expenditures, have no significant overall effect in terms of promoting the high-quality development of resource-based cities. Other policy tools need to be matched to achieve the complementary advantages of various policy expenditures and realize the high-quality development of resource-based cities.

Fifthly, the policy of low energy consumption per unit output value and the high environmental protection expenditure in the environmental priority mode lead to a sharp decline in environmental pollution and an increase in the total population of resource-based cities, and the values of the two observation variables are superior to those in the other scenarios. The accelerated development mode adjusts the above two parameters to the same standard; the values of the environmental pollution and total population variables are slightly inferior to those in the environmental priority mode, while the values of resource consumption are at the optimal level among all scenarios. The reason for this is that the government and enterprises increase their investment in innovation policies, and innovative talent and enterprises develop rapidly, which extensively promotes the output value and industrial added value of high-tech industries in resource-based cities. At the same time, the level of innovation drives the development of new energy and improves energy conversion efficiency, which promotes the level of urban innovation, the value of the output, and the added value of high-tech industries more than that of the environmental priority mode, and the consumption of resources is at the lowest level among all scenarios. This shows that the high industrial added value of the accelerated development mode is obtained at the expense of some environmental benefits, leading to higher environmental pollution than in the environmental priority mode, and the reduction in living environment quality affects the growth of the total population. Comparing the environmental priority mode with the education priority mode, it can be seen that an increase in investment in environmental protection policies in resource-based cities can promote a reduction in resource consumption and the improvement of environmental pollution; the improvement of the urban ecological environment significantly promotes the growth of the total population.

## 5. Discussion

As a strategic guaranteed basis for national energy and resources, relying on innovation to achieve high-quality development is the main direction of future policy regulation for resource-based cities. This paper combines macro- and micro-factors, constructs a high-quality development system driven by innovation in resource-based cities from a system perspective, and takes Fuxin City as an example to simulate the influence of different intensities of policy implementation on the high-quality development of resource-based cities. The result shows that the resource, economic, social, and environmental policies implemented by resource-based cities to achieve innovation-driven development can promote high-quality development. Our findings are in accordance with those of Guo et al. [21], who pointed out that the regional competitiveness of environmental resources, the level of economic development, and the investment in environmental improvement had a significant impact on the sustainable development of resource-based cities. However, they did not demonstrate the impact on high-quality economic development. Using innovative policy tools will produce a synergy among the subsystems of the high-quality development of resource-based cities. An imbalance in the government’s internal policy input to each subsystem will lead to a significant reduction in the effect of policy input and a waste of resources. Only by rationally allocating various policy inputs can policy tools complement each other’s advantages and generate the best benefits from policy input. This is in line with the theoretical framework for the construction of a regional advantage, as Asheim et al. [36] demonstrated that knowledge spillovers across complementary sectors, platform policies based on related variety, and differentiated knowledge bases facilitate economic development within and between regions.

The high-quality development of resource-based cities does not harm the ecological environment. The innovation-driven high-quality development system of resource-based cities is a system involving the coordinated development of the resource, economic, social, and environmental systems. The growth of economic indicators cannot be at the expense of the urban ecological environment; only the coordinated development of various subsystems can ensure the sound development of the high-quality development systems of resource-based cities. Our findings are consistent with those of Zhou et al. [37], who implied that there was no conflict between the implementation of environmental protection and economic growth; the tools with which we effectively handle the relationships between environmental protection, technological innovation, and economic growth will have a major impact on sustainable development. Simulating the high-quality development system of resource-based cities is a dynamic process in which the parameters must be continuously adjusted according to the operation and policy changes of the actual system. Some limitations must be noted. When setting the boundaries of the high-quality development system model for resource-based cities, some secondary influencing factors are excluded due to the consideration of model simplification. Since some parameter values are not available and some exogenous variables are subjective, this affects the simulation results of the model. The model should be revised and improved in the future.

## 6. Conclusions and Policy Implications

### 6.1. Conclusions

In this work, we devised a system dynamics model to simulate innovation-driven high-quality development policy scenarios of resource-based cities from 2008 to 2018 using the data of Fuxin City. The findings provide a valuable scientific reference for the high-quality development of relevant policy design by the governments of resource-based cities.

Innovation policy does not effectively drive high-quality development in the baseline scenario, leading to a low overall level of high-quality development in resource-based cities. The policy of increasing investment in innovation implemented by governments and guiding the governments, enterprises, and universities to participate in innovation can effectively drive the high-quality development of resource-based cities.

The accelerated development mode, which has the highest investment in innovation, has a significant driving effect on the economic growth of resource-based cities. However, it damages the ecological environment, which is not conducive to the ecological environment construction of resource-based cities or the improvement of people’s livelihoods and well-being.

The environmental priority mode is the most appropriate one for the innovation-driven high-quality development of resource-based cities, and it appropriately enhances the input of innovation policies and rationally allocates them within the system, promotes economic and social development, reduces resource consumption, and improves the sustainable development of the ecological environment.

### 6.2. Policy Implications

Based on the research conclusions, we put forward some implications in designing high-development policies for the governments of resource-based cities.

Governments should increase their investments in innovation policies and enhance the driving force of innovation. It is necessary to guide the optimization of the energy industry structure, develop new energy according to local conditions, increase financial investment in science and technology to encourage independent research and development and innovation by universities and enterprises, moderately increase investment in environmental protection and “three waste” treatment, and guide enterprises to promote cleaner production.

The governments should guide the rational and orderly flow of innovation elements and comprehensively promote the efficiency of resource allocation. Resource-based cities should establish an innovative service information platform; encourage scientific and technological innovation research and development through R&D tax incentives, financial subsidies, etc.; cultivate and introduce creative human resources in combination with urban development strategies and industrial planning; guide creative elements to flow to high-tech industries and service industries; promote the rational distribution of creative features and improve resource allocation efficiency; and thus drive their high-quality development.

The government should improve the system construction, strengthen the implementation of relevant rules and regulations, and provide institutional guarantees for the high-quality development of resource-based cities. For example, they could establish and improve the transfer system of resource use rights, the resource income distribution system, the ecological environment compensation system, the green GDP accounting system, the green innovation incentive system, etc., and guide and standardize the behavior of relevant responsible subjects through rules and regulations to promote the high-quality development of resource-based cities. Resource-based cities should implement the concept of “lucid waters and lush mountains are invaluable assets.” Continuous investment in energy conservation and environmental protection innovation policies can effectively maintain the delicate operation of urban high-quality development systems and form positive feedback driven by innovation from resource and environmental subsystems to economic and social subsystems. The government should support the innovation of green technologies and promote clean, low-carbon, safe, and efficient energy use. It is necessary to add environmental evaluation factors into the economic and social evaluation system and promote the healthy operation of each innovation-driven high-quality development subsystem of resource-based cities.

## Figures and Tables

**Table 1:** The composition of an innovation-driven high-quality development system for resource-based cities.

Master System	Subsystem	System Elements
Innovation-driven high-quality development system of resource-based cities	Resource subsystem	Resource consumption, resource demand, energy consumption per unit output value, new energy development, energy conversion efficiency, resource demand factor
	Economic subsystem	GDP, value added of industry, the output value of new and high-technology industries, level of innovation, government expenditure, expenditure on science and technology, number of scientific research achievements
	Social subsystem	Total population, labor inflow, population quality, number of graduate students, scientific and technological human resources, employment rate, education expenditure, etc.
	Environmental subsystem	Waste gas discharge, wastewater discharge, solid waste discharge, environmental protection expenditure, environmental carrying capacity, pollution discharge

**Table 2:** Results of the model tests.

Year	Resource Consumption (tons) Actual Value Simulated Value		Relative Error	Total Population (Ten Thousand People) Actual Value Simulated Value		Relative Error
2008	345	345.0	0.000	192.5	192.5	0.000
2009	357	345.4	−0.032	192.4	191.9	−0.003
2010	367	347.2	−0.054	192.4	191.1	−0.007
2011	381	350.7	−0.080	192.1	190.0	−0.011
2012	370	356.2	−0.037	191.6	188.7	−0.015
2013	346	364.2	0.053	191.1	187.3	−0.020
2014	318	322.8	0.015	191.0	185.9	−0.027
2015	263	286.5	0.089	189.5	184.5	−0.026
2016	237	254.6	0.074	188.9	183.1	−0.031
2017	210	226.6	0.079	186.2	181.7	−0.024
2018	217	201.9	−0.070	185.0	180.4	−0.025

**Table 3:** The parameters of the scenarios.

Primary Parameters	Energy Consumption per Unit of Output Value	Proportion of Internal R&D Expenditure	Proportion of Expenditure on Science and Technology	Proportion of Expenditure on Education	Proportion of Expenditure on Environment
Baseline scenario	0.000078	0.0067	0.0049	0.14	0.0345
Steady development mode	0.000052	0.0075	0.0055	0.20	0.0445
Technology-driven mode	0.000052	0.0080	0.0060	0.20	0.0445
Education priority mode	0.000052	0.0075	0.0055	0.25	0.0445
Environmental priority mode	0.000040	0.0075	0.0055	0.25	0.0545
Accelerated development mode	0.000040	0.0080	0.0060	0.25	0.0545

**Table 4:** Average annual growth rates of the observation variables from 2008 to 2035 unit: %.

Primary Parameters	Resource Consumption	Innovation Level	High-Tech Industrial Output Value	Industrial Added Value	Total Population	Environmental Pollution Degree
Baseline scenario	−2.47	10.50	40.40	8.35	−0.17	−1.11
Steady development mode	−2.66	13.14	47.05	9.72	−0.06	−1.76
Technology-driven mode	−2.87	15.16	55.56	11.48	−0.10	−1.64
Education priority mode	−2.66	13.14	47.05	9.72	−0.04	−1.76
Environmental priority mode	−3.01	13.98	52.61	10.22	0.05	−2.56
Accelerated development mode	−3.23	16.25	59.60	12.55	0.02	−2.46

## Data Availability

Not applicable.
